# Ionization Constants of the Six Dichloroanilines and the Six Dichlorophenols in Aqueous Solution at 25 °C

**DOI:** 10.6028/jres.068A.015

**Published:** 1964-04-01

**Authors:** R. A. Robinson

## Abstract

The thermodynamic ionization constants of the six dichloroanilines and the six dichlorophenols in aqueous solution at 25 °C have been determined by the spectrophotometric method. The *pK* values found are recorded in table 3.

An approximately linear relation is found to exist between the *pK_A_* value of a dichloroaniline and the *pK_P_* value of the corresponding dichlorophenol. The relation is

$$pK_{A}=-9.047+1.401\;pK_{P}.$$

This equation yields *pK_A_* values which differ from the observed by not more than 0.06 *pK* unit and, on the average, by 0.03 *pK* unit; it applies even when both substituents are in the ortho position.

## 1. Introduction

It has been shown [1]^1^ that the *pK* values of the dinitrophenols cannot be predicted with accuracy from data for phenol and the mononitrophenols, if at least one of the nitro groups is in the ortho position with respect to the hydroxyl. The values of phenol, *o*-nitrophenol, *m*-nitrophenol, and *p*-nitrophenol at 25 °C are 9.998 [2], 7.230 [3], 8.355 [4], and 7.156 [5], respectively. Thus, the decrement in *pK* value for *o-, m*-, and *p*-nitro substitution is 2.768, 1.643, and 2.842, respectively. If the effects of substitution were strictly additive, a dinitrophenol without ortho substitution, such as 3,5-dinitrophenol, should have a *pK* value of 6.712; a value of 6.69_2_ has been found [1], a value not seriously different from that predicted. However, with one ortho substituent, as in 2,4-dinitrophenol, a *pK* value of 4.388 is predicted, whereas the experimental value is 4.090 [6], a value considerably different from the predicted. But if both substitutents are in the ortho position, as in 2,6-dinitrophenol, the calculated *pK* value is only 4.462 compared with the observed value of 3.713 [7], the difference being the largest found in the series of dinitrophenols.

It might be expected that additivity relations would have greater validity in the dichlorophenols but there is little data available to confirm this. Judson and Kilpatrick [8] found *pK* 7.850 for 2,4- dichlorophenol; the values of 8.527 for *o*-chlorophenol [9] and 9.418 for *p*-chlorophenol [10] give a predicted value of 7.947.

The *pK* values of the six dichloroanilines and the six dichlorophenols have now been measured in order to test this additivity proposition and, further to see if the Hammett relation [11] could be extended to these disubstituted compounds.

## 2. Experimental Procedure

### 2.1. Materials

The dichloroanilines and dichlorophenols were obtained as commercial products. Details of their recrystallization, melting point, maximum extinction coefficients in alkaline solution (*ϵ*_2_) and in acid solution (*ϵ*_1_) as well as the extinction coefficient at an isosbestic point (*ϵ*) are as follows: 2,3-Dichloroaniline, three times from heptane, mp 23.5–24°, *ϵ*_2_ 2,110 at 292 m*μ, ϵ*_1_ 270 at 269 m*μ* and 220 at 277 m*μ.* 2,4- Dichloroaniline, three times from petroleum ether, mp 50–50.5°, *ϵ*_2_ 2,060 at 297 m*μ*, *ϵ*_1_ 260 at 270 m*μ* and 200 at 279 m*μ.* 2,5-Dichloroaniline, three times from petroleum ether, mp 50–50.5°, *ϵ*_2_ 2,560 at 294, *ϵ*_1_ 400 at 277 m*μ* and 360 at 280 m*μ.* 2,6-Dichloroaniline, three times from heptane, mp 38.5–39°, *ϵ*_2_ 2,570 at 292 m*μ, ϵ*_1_ 280 at 268 m*μ* and 340 at 277 m*μ.* 3,4-Dichloroaniline, dissolved in methanol, precipitated by addition of water and recrystallized three times from petroleum ether, mp 72°, *ϵ*_2_ 1,680 at 297 m*μ, ϵ*_1_ 350 at 271 m*μ* and 320 at 279 m*μ.* 3,5-Dichloroaniline, three times from heptane, mp 50–50.5°, *ϵ*_2_ 1,760 at 294 m*μ, ϵ*_1_ 190 at 272 m*μ* and 180 at 278 m*μ.* 2,3-Dichlorophenol, once from heptane and three times from petroleum ether, mp 58°, *ϵ*_2_ 3,870 at 297 m*μ, ϵ*_1_ 1,840 at 276 m*μ* and 1,790 at 283 m*μ, ϵ*_1_ 1,140 at 267 m*μ* and 1,790 at 278 m*μ.* 2,4-Dichlorophenol, three times from petroleum ether m*μ* 43–43.5°, *ϵ*_2_ 3,620 at 307 m*μ, ϵ*_1_ 2,120 at 284 m*μ, ϵ* 850 at 270 m*μ* and 1,900 at 291 m*μ.* 2,5-Dichlorophenol, twice from petroleum ether, mp 58.5°, *ϵ*_2_ 4,500 at 301 m*μ, ϵ*_1_ 2,360 at 280 m*μ* and 2,160 at 288 m*μ, ϵ* 650 at 264 m*μ* and 2,100 at 284 m*μ.* 2,6-Dichlorophenol, once from methanol-water and then from petroleum ether, mp 66°, *ϵ*_2_ 4,950 at 300 m*μ, ϵ*_1_ 1,940 at 277 m*μ* and 1,900 at 283 m*μ ϵ* 800 at 265 m*μ* and 1,750 at 281 m*μ*. 3,4-Dichlorophenol, three times from petroleum ether, mp 66°, *ϵ*_2_ 2,830 at 303 m*μ*, 1,780 at 284 m*μ*, *ϵ* 1,000 at 274 m*μ* and 1,650 at 288 m*μ*. 3,5-Dichlorophenol, three times from heptane, mp 67°, *ϵ*_2_ 3,220 at 297 m*μ*, *ϵ*_1_ 1,570 at 277 m*μ* and 1,560 at 284 m*μ*, *ϵ* 700 at 267 m*μ* and 1,420 at 279 m*μ*.

**Figure 1:**
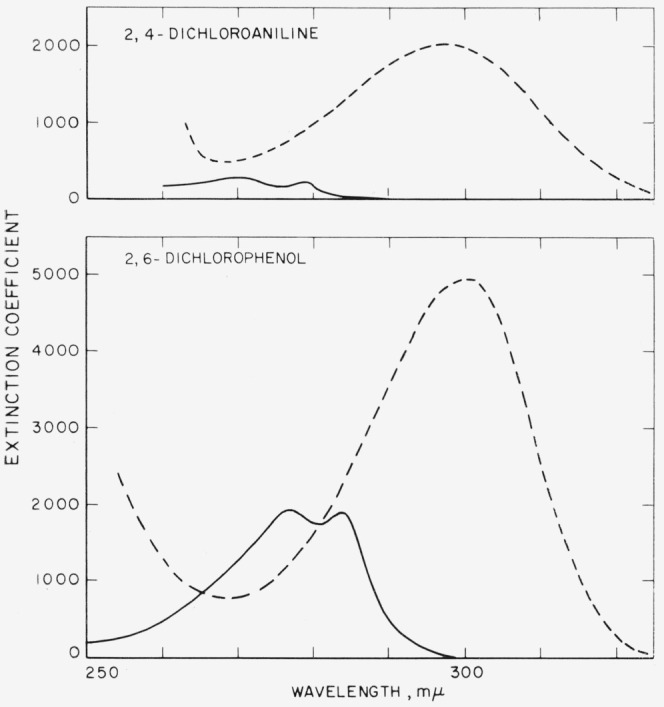
Spectral absorption curves of 2,4-dichloroaniline and 2,6-dichlorophenol in aqueous solution at 25 °C ----, With addition of alkali. ——, With addition of acid.

Absorption spectra were measured either on a Cary or on an Optica instrument. Two typical plots of extinction coefficient versus wavelength are shown in figure 1. It will be noted that the plot for the aniline resembles that of the phenol, the maxima in acid and alkaline solution being found at approximately the same wavelength. Extinction coefficients are, however, much lower for the anilines than for the phenols; moreover, while there are well defined isosbestic points for the phenols, there are none for the anilines in the range of wavelength studied in this paper.

### 2.2. Determination of *pK* Values

The spectrophotometric method followed in determining the *pK* values was similar to that used by Bates and Schwarzenbach [12] and by Robinson and Biggs [13]. Since the anilines have *pK_A_* values between 0.4 and 3, solutions were prepared of each aniline in hydrochloric acid solution of concentration comparable with the ionization constant of the aniline so that the concentration ratio of aniliniumion, HR^+^, to uncharged aniline, R, lay between 0.2 and 0.8. The *pK_A_* value of the aniline 2 is then given by

$$pK_{A}=-\log\;\left[H^{+}\right]-\log\;\frac{D-D_{1}}{D_{2}-D}-\log\;\frac{\gamma_{H}+\gamma_{R}}{\gamma_{\text{HR}}+}$$ (1)

where the symbols have their usual meaning. Thus, *D*_1_ is the optical density of a solution of the aniline to which sufficient concentrated hydrochloric acid has been added to convert the aniline entirely into the positively charged anilinium ion, *D*_2_ the optical density of the solution to which sodium hydroxide has been added and the aniline exists entirely in the uncharged form, and *D* is the optical density of the solution to which dilute hydrochloric acid has been added and the aniline is present partly in the charged and partly in the uncharged form; in each of the three solutions, the stoichiometric concentration of aniline is the same. γ denotes an activity coefficient and [H^+^] the hydrogen ion concentration. The latter was taken as the stoichiometric concentration of hydrochloric acid less the amount of acid required to form the anilinium ion and was expressed on a molarity basis, i.e., in moles per liter; the ionization constant, *K_A_*, is therefore also on the molarity scale. As the ionization constants on the molarity and the molality scale are related by an expression including the density of the solvent, they differ little if the solvent is water. The *pK_A_* values recorded later are on the molarity scale but those on the molality scale would be only 0.001 lower. It is assumed that the last term in eq (1) is negligible; with one exception, this seems justified by the constancy of the *pK* values obtained over a range of acid concentration; the exception is 2,6-dichloroaniline to which further reference will be made below.

For the phenols, with *pK_P_* values between 6.8 and 8.6, buffer solutions were used. One of these was the equimolal (*m*) mixture of potassium dihydrogen phosphate and disodium hydrogen phosphate whose total ionic strength is *I*=4 *m* (this buffer is referred to as phosphate buffer). The other was a mixture of tris-(hydroxymethyl)-aminomethane (*m*_1_) and its hydrochloride (*m*_2_) with *m*_1_/*m*_2_= 1.0157 and *I=m_2_* (referred to as tris buffer). The *pK* of the phenol is given by

$$pK_{P}=-\log\;\left[a_{H}+\gamma_{{\text{Cl}}^{-}}\right]-\log\;\frac{D-D_{1}}{D_{2}-D}+\log\;\frac{\gamma_{\text{HR}}+\gamma_{{\text{Cl}}^{-}}}{\gamma_{R^{-}}}.$$ (2)

Again it is assumed that the last term is negligible and this seemed to be valid for all six phenols, provided that the total ionic strength of the buffer solution did not exceed 0.1. This point was studied in some detail in the case of 2,6-dichlorophenol, measurements being made in eleven phosphate buffer solutions whose ionic strengths lay between 0.01 and 0.20. The results in table 2 for this dichlorophenol indicate that the apparent *pK* values derived from measurements in solutions of *I*>0.10 are slightly but significantly lower than those derived from measurements in more dilute solutions. Thus, not surprisingly, the final term in eq (2) is not negligible if the ionic strength of the medium exceeds 0.1. In deriving a mean *pK* value for this dichlorophenol (table 2), the *pK* values at *I*=0.12, 0.15, and 0.20 were excluded; for the other dichlorophenols no measurements were made at any ionic strength exceeding 0.1. Values of $-\log\;\left(a_{H^{+}}\gamma_{{\text{Cl}}^{-}}\right)$ were taken from the paper of Bates and Gary [14] as follows:

**Table:** 

*I*	0.01	0.02	0.03	0.04	0. 05	0.06	……
Phosphate	7.111	7.080	7.058	7.040	7.026	7.013	……
Tris	8.176	8. 207	8.232	8.251	8. 266	8.280	……
*I*	0.07	0.08	0.09	0.10	0.12	0.15	0. 20
Phosphate	……	6.992	……	6.974	6.959	6.940	6. 912
Tris	8.292	8.302	8.312	8.321	……	……	……

### 2.3. Results

Table 1 gives data for the six dichloroanilines. The concentration of aniline, the length of the absorption cells, the wavelength at which optical density measurements were made, *D*_1_ and *D*_2_ are recorded first and then values of *D* and *pK_A_* (calculated by eq (1)) are given for a number of solutions of hydrochloric acid of different concentration.

Table 2 gives similar data for the six dichlorophenols, except that the nature of the buffer mixture is recorded and the first column refers to the total ionic strength of the buffer solution. For 2,6- dichlorophenol, measurements were made at two wavelengths, with concordant results. For 2,3-dichlorophenol, measurements were made with each of the buffer solutions, again with concordant results. Each *pK* value is corrected for the influence of the phenol on the $\log\;\left(a_{H^{+}}\gamma_{{\text{Cl}}^{-}}\right)$ value of the buffer solution [15].

The measurements recorded in these tables were made using specimens of aniline or phenol which showed no change in *pK* on further recrystallization.

## 3. Discussion

### 3.1. 2,6-Dichloroaniline

Inspection of table 1 shows that constant *pK_A_* values are obtained, independent of the hydrochloric acid concentration, except in the case of 2,6-dichloroaniline. The 2,6-dichloroanilinium ion is a comparatively strong acid and considerable amounts of hydrochloric acid had to be added to get favorable optical density readings; it is not surprising that, at such high ionic strengths, the last term of eq (1) is no longer negligible. A similar dependence of apparent *pK* on hydrochloric acid concentration was found with *o*-nitroaniline [10]. The data were therefore fitted to the equation

$$pK_{A}\;\left(\text{apparent}\right)=pK_{A}+aC_{\text{HCl}}$$ (3)

and the true *pK_A_* value obtained as the limiting value of *pK_A_* (apparent) when *C*_HCl_=0; the method of least squares gave *pK_A_*=0.422, *a*=0.120 liter mole^−1^.

It is worth while considering the reason for this variation in the activity coefficient term of eq (1). For this purpose, we rewrite it as log $\left(\gamma_{\text{HCl}}^{2}\gamma_{R}/\gamma_{H}^{2}{\;}_{\text{RCl}}\right)$. Here γ_HCl_ is the mean ionic activity coefficient of hydrochloric acid in the presence of a small amount of dichloroaniline; this can, without serious error, be taken as the activity coefficient in aqueous solution, values of which have been tabulated [16]. γ_HRCl_ is the activity coefficient of the hydrochloride of the dichloroaniline at low concentration in the presence of comparatively large amounts of hydrochloric acid. This has not been measured but we may speculate that the behavior of the activity coefficient of an aniline hydrochloride might be similar to that of ammonium chloride, which is known to resemble that of potassium chloride [17]. The activity coefficient might then be calculated by the equation

$$\log\;\gamma_{\left(0\right)\text{KCl}}=\log\;\gamma_{\text{KCl}\left(0\right)}-\alpha_{2}m,$$ (4)

a relation sometimes known as Harned’s rule. Here γ_(0)KCl_ is the activity coefficient of potassium chloride present in vanishingly small concentration in a solution of hydrochloric acid of concentration *m*, γ_KCl(0)_ is the activity coefficient of potassium chloride in aqueous solution at the same concentration, *m*, and α_2_ is a parameter which depends on the total ionic strength of the solution. At *m*=0.5, log γ_KCl(0)_ =−0.1876, α_2_= —0.07 [18] and hence log γ_(0)KCl_ = −0.1526. log γ_HCl_ is known to be −0.1209 at *m*=0.5 and hence log $\left(\gamma_{\text{HCl}}^{2}\gamma_{R}/\gamma_{\text{HRCl}}^{2}\right)=0.063$, assuming γ_R_=1. This assumption that the activity coefficient of an uncharged species can be equated to unity is supported by some recent experimental evidence; thus, it has been found [19] that the activity coefficient of mannitol present in very small amount in 0.5 *M* sodium chloride solution is only 0.993, that of urea in the same solution [20] is 0.983.

Now log $\left(\gamma_{\text{HCl}}^{2}\;\gamma_{R}/\gamma_{\text{HRCl}}^{2}\right)$, which we have calculated to be 0.063, should by eq (3) represent the difference between the true *pK_A_* which has been found to be 0.422 and the apparent *pK_A_* at *m*=0.5 which, by interpolation in table 1 or by eq (3), is 0.482. The difference in *pK_A_*, 0.060, compares with 0.063 calculated. The agreement is better than one would expect in view of the approximation that had to be made but it does confirm that the variation in apparent *pK_A_* is not unreasonably large.

### 3.2. p*K* Values and Comparison With Other Data

Values of *pK* for the dichloroanilines and the dichlorophenols (with the standard deviations in parentheses) are collected in table 3.

Some previous measurments of these ionization constants have been found in the literature. For 2,5-dichloroaniline Gillois and Rumpf [21] found *pK_A_* 1.57 compared with 1.52_9_ in this work. In the case of 2,4-dichloroaniline, whereas the present work gives 2.01_6_, Paul [22] gave a tentative value of 2.00. Högfeldt and Bigeleisen [23] found 2.05 at 22 °C and Brønsted et al. [24] found 2.14 at 21°. Judson and Kilpatrick [8] gave 7.85_0_ for 2,4-dichlorophenol and quote 7.75 [25] and 7.89 [26] from the literature; the present value is 7.89_2_. 2,6-Dichlorophenol has been studied by the same spectrophotometric method [27] but with a succinate-phosphate buffer solution; a *pK* value of 6.786 was found. Murray and Gordon [25] studied all six of the dichlorophenols by electrometric measurements in a water-methanol solvent and applied a correction to give data in aqueous solution by measuring phenol itself in both solvents and assuming that the difference in *pK_P_* value could be applied to the dichlorophenols. As they found *pK_P_* 9.78 for phenol in aqueous solution, it may be that they overcorrected their data by about *pK_P_* 0.22. If their values are raised by this amount, agreement is found with the values in table 3 to within 0.02 to 0.07 *pK_P_* units, except for 2,6- dichlorophenol for which their adjusted value is 7.01.

Table 3 also contains values of Δ*pK*(1) *≡ pK* (calc.)— *pK* (obs.), the calculated values being obtained from *pK* values for monosubstituted compounds with the assumption of additivity of the effects of substituent groups. Data for the dinitrophenols are given for comparison.^3^ It will be seen that, as mentioned in the introduction, large deviations from additivity are noticed in the 2,3- and 2,6-dinitrophenols, i.e., if both nitro groups and the hydroxy group are adjacent to one another. The additivity principle holds much better in the dichlorophenols and the dichloroanilines—only in the 2,6-compounds where both substituents are ortho to the hydroxy or amine group is there marked departure from additivity.

### 3.3. Hammett Relation

This relation can be expressed by the equation

ΔpK=ρσ

where Δ*pK* is the difference between the *pK* value of a parent acid and that of one of its substituted derivatives, *p* is a characteristic of the parent acid and, by convention, is taken as unity for benzoic acid; *σ* is a characteristic of the substituent group or groups.

Jaffé [29] has assigned values of 0.600 and 0.746 to the Hammett *σ* parameter fol 3,4- and 3,5-dichloro substitution, respectively. However, more recent determinations [30, 31] of the ionization constants of 3,4- and 3,5-dichlorobenzoic acid give 3.64 and 3.43, respectively; these lead to 0.56 and 0.77 for 3,4- and 3,5-dichloro substitution, respectively.

Biggs and Robinson [10] measured the ionization constants of a number of monosubstituted anilines and phenols; they expressed the Hammett relation for substituted anilines by the equation

$$pK_{A}=4.580-2.889\sigma$$ (5)

and for substituted phenols by

$$pK_{P}=9.919-2.229\sigma .$$ (6)

Substituting the above values of *σ* in these equations, we calculate *pK* 2.96 and 2.36 for 3,4- and 3,5-dichloroaniline respectively and 8.67 and 8.20 for 3,4- and 3,5-dichlorophenol, respectively. These differ from the observed values by 0.03, 0.02, 0.08, and 0.01, respectively; the agreement between observed and calculated values is comparable to that found in a series of monosubstituted compounds.

### 3.4. A Linear Relation Between the *pK* Values of the Dichloroanilines and Those of the Dichlorophenols

A simple rearrangement of eq (5) and eq (6) with elimination of the *σ* term leads to a relation between the *pK_A_* value of a monosubstituted aniline and the *pK_P_* value of the corresponding monosubstituted phenol; this takes the form

$$pK_{A}=-8.275+1.296\;pK_{P}.$$ (7)

**Figure 2:**
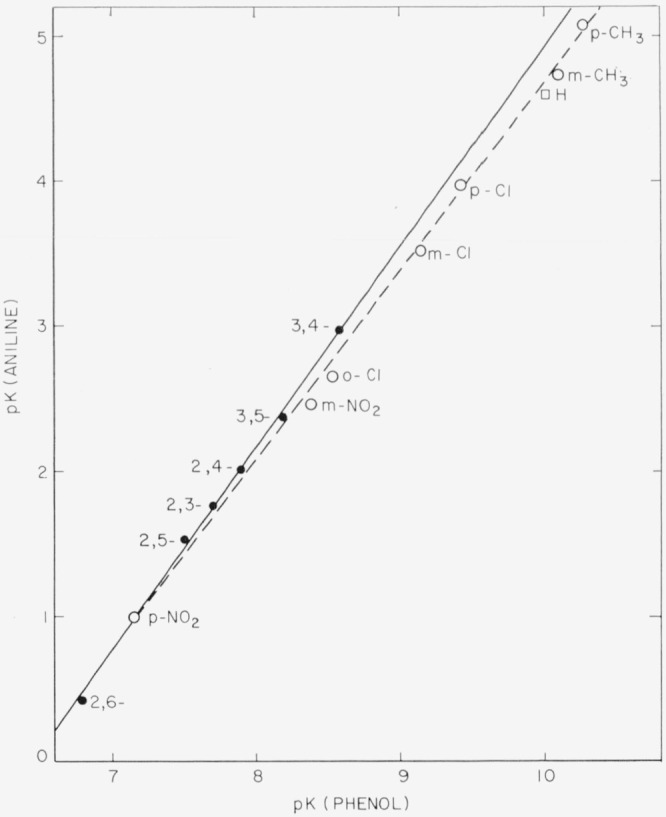
*pK_A_* of an aniline versus *pK_P_* of the corresponding phenol □, Aniline-phenol ○, *o-, m*-, and *p*- methyl, *o*-, *m-, p*- chloro, and *m*- and *p*-nitroanilines and phenols. ●, Dichloroanilines and phenols. ----, Line of the eq (7)*pK_A_ = −*8.275+1.296 *pKp* for monosubstituted anilines and phenols. ——, Line of the eq (8)*pK_A_*=−9.047+1.401 *pKp* for dichloroanilines and phenols.

Figure 2 is a plot of this equation with points for (1) unsubstituted aniline and phenol and (2) some monosubstituted compounds. Equation (7) is merely a restatement of the Hammett relation avoiding direct reference to substituted benzoic acids.

Figure 2 also contains points for the six dichloroanilines and dichlorophenols which form the subject of this study. It will be seen that the points for the dichloro compounds lie close to a straight line whose slope is nearly but not exactly that of the line for the monosubstituted compounds. By the method of least squares, the best equation for the line through the points for the disubstituted compounds was found to be

$$pK_{A}=-9.047+1.401\;pK_{P}.$$ (8)

Table 3 gives values of Δ*pKA*(2) *≡ pK_A_* (calc.)−*pK_A_* (obs.) where *pK_A_* (calc.) is obtained by eq (8). The average value of Δ*pK_A_* (2) is 0.032. Thus the linear relation between *pK* values of dichloroanilines and dichlorophenols holds somewhat better than the corresponding linear relation for monosubstituted compounds. What is indeed remarkable is that a linear relation is valid in a series of six dichloro compounds in four of which at least one substituent is in the ortho position. Further study will be needed to ascertain the validity of this linear relation in other disubstituted compounds but there are sufficient data available to show that there are limits. Thus, we have *pK*= −1.03 for 4-chloro-2-nitroaniline [32] and 6.46 for the corresponding phenol [33]; eq (8) predicts *pK*= 0 for this aniline. Again, we have *pK* 4.57 for 2,5-xylidene [34] and 10.40 for 2,5-xylenol [9]; eq (8) predicts *pK* 5.52 for 2,5-xylidene. It is clear eq (8) is not valid for 4-chloro-2-nitro substitution nor for the dimethyl compounds, the discrepancy between observed and predicted *pK* values being 1.03 in the former case and 0.95 in the latter. There is, however, one indication that the validity of eq (8) is not limited to dichloro substitution; Wepster [35] has found *pK* 1.50 for 4-nitro-*m*-toluidine; *pK* 7.409 has been found for 4-nitro-*m*-cresol [3] and substitution of this value in eq (8) gives *pK* 1.33 for 4-nitro-*m*-toluidine, a value only 0.17 below the observed. Further work will be necessary to reveal the applicability of eq (8) to compounds other than the dichloroanilines and phenols.

## Figures and Tables

**Table 1:** Ionization constants of dichloroanilines in aqueous solution at 25°

Conc. of HCl	*D*	*pK*

2,3-Dichloroaniline 4.36×10^−4^ *M* 1 cm 292 m*μ D*_1_ 0.002, *D*_2_ 0.922		

0.004312	0.743	1.762
.005854	.697	1.755
.01004	.591	1.757
.01560	.490	1.762
.01723	.467	1.763
.02001	.430	1.762
.02479	.385	1.755
.03003	.340	1.763
.03993	.280	1.766
.05005	.237	1.769

2,4-Dichloroaniline 3.52×10^−4^ *M* 1 cm 297 m*μ* D_1_ 0, *D*_2_ 0.725		

0.002513	0.578	2.010
.003518	.535	2.015
.005025	.480	2.017
.007035	.424	2.013
.008894	.380	2.017
.01005	.356	2.023
.01158	.332	2.017
.01508	.286	2.014
.01849	.248	2.022

2,5-Dichloroaniline 3.75×10^−4^ *M* 1 cm 294 m*μ D*_1_ 0, *D*_2_ 0.959		

0.007189	0.773	1.529
.01055	.710	1.526
.01524	.633	1.533
.01963	.580	1.525
.03007	.478	1.528
.03994	.409	1.530
.04460	.383	1.531
.05076	.352	1.532

2,6-Dichloroaniline 1.167×10^−4^ *M* 4 cm 202 m*μ D*_1_ 0, *D*_2_1.200		

0.1748	0.807	0.445
.2328	.724	.451
.2970	.650	.454
.3365	.604	.467
.3544	.593	.461
.4071	.549	.464
.5192	.463	.487
.5862	.425	.493

3,4-Dichloroaniline 4.76×10^−4^ *M* 1 cm 297 m*μ D*_1_ 0, *D*_2_ 0.802		

0.0002023	0.705	2.977
.0004938	.598	2.962
.0006912	.536	2.969
.001002	.462	2.964
.001248	.411	2.973
.001507	.373	2.963
.001752	.339	2.966
.001977	.311	2. 971
.002513	.266	2.963

3,5-Dichloroaniline 1.21×10^−4^ *M* 4 cm 294 m*μ D*_1_ 0, *D*_2_ 0.850		

0.001421	0.634	2.389
.001991	.578	2.383
.002978	.498	2.382
.003341	.474	2.382
.003710	.451	2.384
.004460	.411	2.386
.004972	.391	2.379
.005938	.354	2.378
.007132	.313	2.386
.007711	.300	2.380

**Table 2:** Ionization constants of dichlorophenols in aqueous solution at 25°

*I*	*D*	*pK* (corr.)

2,3-Dichlorophenol 1.03×10^−4^ *M* 4 cm 297 m*μ D*_1_ 0.006, *D*_2_ 1.594 Phosphate buffer		

0.01	0.331	7.696
.02	.318	7.689
.03	.303	7.694
.04	.292	7.696
.05	.286	7.694
.06	.277	7.699
.08	.270	7.691

4.98×10^−5^ *M* 4 cm 297 m*μ D*_1_ 0.003, *D*_2_ 0.769 Tris buffer		

0.02	0.590	7.689
.03	.596	7.696
.04	.602	7.694
.06	.609	7.702
.07	.611	7.707
.09	.617	7.706
.10	.625	7.687

2,4-Dichlorophenol 6.26×10^−5^ *M* 4 cm 307 m*μ D*_1_ 0.007, *D*_2_ 0.907 Tris buffer		

0.01	0.599	7.889
.02	.611	7.895
.03	.627	7.886
.04	.629	7.900
.05	.640	7.890
.06	.644	7.895
.07	.649	7.891
.08	.654	7.894
.09	.660	7.890
.10	.666	7.884

2,5-Dichlorophenol 4.64×10^−5^ *M* 4 cm 301 m*μ D*_1_ 0.010, *D*_2_ 0.835 phosphate buffer		

0.01	0.244	7.509
.02	.236	7.501
.03	.226	7.507
.04	.220	7.506
.05	.212	7.514
.06	.211	7.504
.08	.200	7.515
.10	.196	7.510

2,6-Dichlorophenol 5.32×10^−5^ *M* 4 cm 240 m*μ D*_1_ 0.036, *D*_2_ 1.338 phosphate buffer		

0.01	0.908	6.792
.02	.889	6.795
.03	.876	6.793
.04	.867	6.791
.05	.858	6.790
.06	.843	6.799
.08	.836	6.788
.10	.823	6.789

5.32×10^−5^ *M* 4 cm 300 m*μ D*_1_ 0.001, *D*_2_ 1.053 Phosphate buffer		

0.01	0.705	6.793
.02	.693	6.790
.03	.684	6.787
.04	.675	6.787
.05	.665	6.791
.06	.657	6.792
.08	.646	6.790
.10	.637	6.789
.12	.634	6.780
.15	.627	6.773
.20	.608	6.777

3,4-Dichlorophenol 5.90×10^−5^ *M* 4 cm 303 m*μ D*_1_ 0.017, *D*_2_ 0.668 Tris buffer		

0.01	0.199	8. 587
.02	.210	8.582
.03	.221	8.573
.04	.225	8.579
.05	.229	8.582
.06	.232	8. 587
.07	.235	8.590
.08	.240	8. 585
.09	.241	8.592
.10	.244	8. 592

3,5-Dichlorophenol 2.17×10^−4^ *M* 1 cm 297 m*μ D*_1_ 0.004, *D*_2_ 0.699 Tris buffer		

0.01	0.344	8.187
.02	.359	8.184
.03	.368	8.184
.04	.374	8.193
.05	.385	8.180
.06	.388	8.188
.07	.394	8.184
.08	.397	8.177
.09	.401	8.186
.10	.405	8.185

**Table 3:** aIonization constants (*pK* values) of dichloroanilines and dichlorophenols in aqueous solution at 25°

	*pK*		Δ*pK*(1)			Δ*pK_A_*(2)
			
	Dichloroaniline	Dichlorophenol	Dinitrophenol	Dichlorophenol	Dichloroaniline	
2,3–	1.76_1_(0.005)	7.69_6_(0.006)	0.65	−0.05	0.16	−0.02_6_
2,4–	2.01_6_(.004)	7.89_2_ (0.005)	.25	.06	.01	−.00_6_
2,5–	1.52_9_(.003)	7.50_8_ (.005)	.40	.15	.03	−.05_7_
2,6–	0.42_2_ (.004)	6.79_1_(.003)	.71	.27	.28	.04_5_
3, 4–	2.96_8_(.005)	8.58_5_ (.006)	.13	−.04	−.07	.01_3_
3, 5–	2.38_3_(.003)	8.18_5_(.004)	.11	.07	.06	.03_7_

a Δ*pK*(1)=*pK* (calc.)−*pK* (obs.), *pK* (calc.) being calculated from *pK* values of the monosubstituted compounds. Δ*pK_A_* (2)*≡pK_A_* (calc.)−*pK_A_* (obs.), *pK_A_* (calc.) being calculated by eq (6).

## Footnotes

1 Figures in brackets indicate the literature references at: the end of this paper.

2 By *K_A_* for an aniline is meant the acidic ionization constant of the corresponding anilinium ion, the equation for whose dissociation is RH^+^→H^+^+R and

$$K_{A}=\frac{C_{H^{+}}C_{R}}{C_{{\text{HR}}^{+}}}\frac{\gamma_{H^{+}}\gamma_{R}}{\gamma_{{\text{HR}}^{+}}}$$

3 The Δ*pK*(1) values recorded for the dinitrophenols differ slightly from those given previously [1]. The change results from the use of more recent *pK* determinations of the mononitrophenols. The value now adopted, 7.230 [3], for *o*-nitrophenol is in good agreement with *pK* 7.234 by Judson and Kilpatrick [8] and 7.229 by Dippy, Hughes, and Laxton [28]. The value of *pK* 8.355 for *m*-nitrophenol [4] is preferred to the earlier value of 8.399 [10] because the more recent measurements were made in a tris buffer in place of the borax buffer of comparatively low *p*H used earlier. Judson and Kilpatrick [8] found *pK* 8.346 for *m*-nitrophenol.
